# Exploring factors affecting Chinese adolescents’ perceived usefulness and engagement with a stress management app: a qualitative study

**DOI:** 10.3389/fpsyg.2023.1249093

**Published:** 2023-11-20

**Authors:** Xiaoyun Zhou, Matthew Bambling, Xuejun Bai, Anthony C. Smith, Sisira Edirippulige

**Affiliations:** ^1^Centre for Online Health, The University of Queensland, Brisbane, QLD, Australia; ^2^Centre for Health Services Research, The University of Queensland, Brisbane, QLD, Australia; ^3^School of Public Health, The University of Queensland, Brisbane, QLD, Australia; ^4^Queensland Centre for Mental Health Research, The University of Queensland, Brisbane, QLD, Australia; ^5^Navitas ACAP School of Psychology, Brisbane, QLD, Australia; ^6^Faculty of Medicine, The University of Queensland, Brisbane, QLD, Australia; ^7^Academy of Psychology and Behavior, Tianjin Normal University, Tianjin, China; ^8^Centre for Innovative Medical Technology, University of Southern Denmark, Odense, Denmark

**Keywords:** adolescents, stress management, mobile health, coping skills, qualitative study, stress, mental health, digital health

## Abstract

**Introduction:**

Providing adolescents with stress management interventions via mobile apps has potential for overcoming barriers to traditional in-person services, such as stigma, cost and travel. However, the effectiveness remains uncertain and engagement level remains low. Therefore, it is essential to understand adolescents’ user experience of such apps, however, such research is scarce. This study aimed to address this research gap by exploring factors affecting Chinese adolescents’ perceived usefulness and engagement of a stress management app, which was developed for them.

**Methods:**

A qualitative study design involving focus group interviews and inductive thematic analysis was adopted. A purposive sampling method was employed, resulting in five focus groups (*n* = 39 adolescents).

**Results:**

Two themes emerged: (1) mechanism and determinants of usefulness and (2) facilitators and barriers to engagement. The app was found to be helpful in managing chronic and simple stressors by promoting positive behavior, cognition, and physical changes. Relevance to real-life situations, peer support, and planning and monitoring features were found to increase usefulness. Participants suggested adding one-on-one chat support for managing acute stressors. Multimedia, logical content arrangement, combining psychoeducation and skills training, gamification, customization, and an appealing user interface were engaging factors for adolescents, whilst text-heavy content, pedagogical and monotonous tones, technical issues were found to disengage adolescents.

**Conclusion:**

Stress management apps should involve simple and evidence-based coping skills training, target adolescents’ real-life problems, promote positive peer influence, address both chronic and acute stressors. Additionally, such apps should have logical arrangement of content, be interactive and customizable, and involve multimedia and gamification features to engage adolescents.

## Introduction

Psychological stress is a major concern for adolescents worldwide, with a profound impact on their mental (McLaughlin and Hatzenbuehler, 2009; Jayanthi et al., 2015; Gomes and Grace, 2017) and physical health (De Vriendt et al., 2009; Gianaros and Wager, 2015). China has an adolescent population of 146 million (UNICEF China and UNFPA China, 2018), and they face significant stress due to academic pressures (Liu and Lu, 2011; Ye et al., 2019), culturally-unique family problems (Xing et al., 2010), and rapid social changes (Xin et al., 2012). For example, Chinese adolescents, like many individuals from collectivist cultures, perceive and experience stress differently due to socio-cultural factors, values, familial expectations, and educational pressures that are uniquely emphasized in Chinese society (Zhou et al., 2023a). Recent studies have found that Chinese adolescents have high prevalence of mental health problems, such as depression and anxiety (Liu et al., 2001; Liu, 2004; Xin et al., 2012; Wang et al., 2014), and stressful life events are a significant contributor (Liu and Tein, 2005). Failing to address these mental health issues can lead to poor academic performance, substance abuse, and even suicide. Another consequence is these mental health issues might extend to adulthood and impair their mental health and productivity as adults. In recent years, the COVID-19 has placed additional pressure on adolescents irrespective of country of origin (Zhou et al., 2020; Panchal et al., 2021; Zhou C. et al., 2021). Therefore, it is essential to prioritize the development and implementation of effective and culturally sensitive interventions to address psychological stress among adolescents globally and in China specifically. However, several factors hinder access to traditional in-person mental health support, including stigma, cost, and travel associated with in-person services (Hunt and Eisenberg, 2010; Chen et al., 2014).

Mobile mental health refers to the use of mobile devices, such as smartphones and tablets, to provide mental health services and information (World Health Organisation, 2011). Mobile mental health has been widely used to manage minor mental health conditions, such as work-related stress among adults (Paganin and Simbula, 2020), more severe mental health issues, such as psychiatric disorders (Melbye et al., 2020), and behavioral problems with young people (Egilsson et al., 2023). Mobile health has become increasingly popular among adolescents due to their accessibility and convenience (Tran et al., 2018; Nicol et al., 2022). Mobile app-based interventions provide a viable solution to the challenges faced by Chinese adolescents, as it costs less, requires less travel, and as these interventions are typically anonymous, they can also reduce stigma (Benjet et al., 2020) and it is more accessible as well. Despite of rapid growth in numbers in mobile mental health apps for adolescents, there is limited research evidence to support the effectiveness of apps for adolescents with mental health problems (Grist et al., 2017; Punukollu and Marques, 2019). User engagement refers to “a category of user experience characterized by attributes of challenge, positive affect, endurability, aesthetic and sensory appeal, attention, feedback, variety/novelty, interactivity, and perceived user control” (Egilsson et al., 2023). Poor user engagement is typically associated with increased drop-out rate (Scherer et al., 2017) and decreased effectiveness of mobile mental health interventions (Donkin et al., 2011).

In addition, engagement levels range widely across mobile app interventions (Zhou X. et al., 2021; Melcher et al., 2022). For example, Bohleber et al. (2016) found that the adolescents did not use the mental health app consistently due to technical difficulties and insufficiently obvious benefits. Similarly, a review conducted by Torous et al. (2018) found that the user engagement of mental health apps is low because they are not user-centred, do not respect privacy, do not address users’ main concerns, lack privacy, are not trustworthy and not effective in emergencies. Understanding adolescents’ experience, perspectives and preferences for mental health apps can help design effective mental health apps and improve engagement and mental health outcomes.

A systematic review explored adolescents’ engagement with digital health interventions and found that adolescents preferred digital health interventions that offer personalized features, videos, limited text, social connectivity and text message reminders (Liverpool et al., 2020). Even though there are studies which investigated adolescents’ perspectives with mental health apps (Tran et al., 2018; Agapie et al., 2022; Nicol et al., 2022), none of the studies investigated adolescents’ experience with stress management apps. This study addresses this research gap by investigating Chinese adolescents’ experience and perspectives with a mobile app, the Coping Camp, which was designed for stress management for Chinese school adolescents (Zhou et al., 2022b).

This study is a post-intervention user experience study following a randomized controlled trial which investigated the efficacy of an app-based intervention for stress management (the Coping Camp) among Chinese adolescents (Zhou et al., 2023b). The app intervention was based on Stress Inoculation Training (Meichenbaum, 1985), comprised of 11 modularized skills training sessions, and lasted for 11 weeks. The objectives of this study were to investigate user experience with and perspectives on the Coping Camp, the factors that impacted perceived usefulness of the app, and factors impacting the perceived engagement of the app. In addition, this study also sought their suggestions for improving usefulness and engagement of the app.

Understanding the perspectives and experience of adolescents who already have experience with stress management apps can provide insights into adolescents’ preferences for stress management apps. As mental health concerns and preferences for mental health services may vary across different countries and cultures (Pramukti et al., 2020), with an exceptional focus on Chinese adolescent population, this study also can provide valuable information for developing culturally sensitive and adolescent-centred mental health apps in similar cultural and societal backgrounds. In addition, as this app intervention was conducted in school settings, the results of this study can also provide insights into developing school-based stress management apps for adolescents.

## Methods

### Study design

This study adopted a qualitative study design (Polit and Beck, 2008) with focus group interviews. The reporting of this study followed Consolidated Criteria for Reporting Qualitative Research (COREQ) (Tong et al., 2007) (see Supplementary Datasheet 1).

This qualitative study was a post-intervention user experience study, which followed a cluster randomized controlled trial (RCT) which evaluated the efficacy of a stress management app, the Coping Camp, among a universal sample in Chinese high school students (Zhou et al., 2022b). More details relating to the Coping Camp app is published previously (Zhou et al., 2023b). The Coping Camp was based on stress inoculation training (SIT) (Meichenbaum, 1985), which was a type of cognitive behavioural therapy (CBT) developed by Meichenbaum with a specific purpose for managing stress and related problems (e.g., anger, burnout). The SIT was tailored to suit Chinese high school students according to our previous exploratory study (Zhou et al., 2023a). The Coping Camp app comprised of psychoeducation, which was delivered via videos, and coping skill training, which was delivered via audios, texts and graphs, and a discussion board which allowed students to share their stress experience and coping strategies and served as peer support.

### Recruitment, sampling and participants

#### Recruitment

Current qualitative study was conducted in February 2021 in two high schools (one private and one public) in Mianyang City, which is a moderate city in terms of size, population and economic development and is located in southwestern China. The principal investigator (XZ) recruited participants from the intervention group (total *N* = 275 students) right after the post-intervention assessment of the RCT study. The cluster RCT study included those aged between 15 and 19 years old, studied at grade 10 and 11, further inclusion criteria in present focus group study only required the participants to be in the intervention group and to have used the app at least for more than once out of 11 sessions. The exclusion of participants was administrated in cluster RCT study. The exclusion criteria included (1) students should not have suicidal ideation; (2) should not be diagnosed with a mental disorder at the time of recruitment; (3) not be taking psychiatric medications and (4) they should not be receiving regular psychotherapies. Additionally, as adolescents, who did not use the app at all after enrollment, were not able provide their experience and perspectives of using the app, they were also excluded from the study.

#### Sampling

A purposive sampling method was adopted. Participants were recruited from three subsets of students: (1) highly engaged students, who attended 9–11 sessions out of 11 sessions in the Coping Camp app; (2) moderately engaged students who attended 4–8 sessions and (3) lowly engaged students who attended 1–3 sessions. In our previous trial, there were 19 (6.9%) students who did not complete any session, and 43 (15.6%) students, 92 (15.6%) students, and 121 (44%) students who were lowly, moderately, and highly engaged participants, respectively. User engagement was verified by one member of the research team (SE) through a filtered search of Coping Camp’s user activity data logs. Overall, the purposeful sampling criteria were intended to identify a sample of participants that was representative of the whole user population. The recruitment strategies included announcement in the classrooms and text messages sent to students’ mobile phones. We aimed to recruit one to two groups from each subset, which resulted in 24–48 students in total. Active recruitment ceased when the data reached saturation.

#### Participants

Potential participants who were interested were provided an information sheet and consent form. The information sheet outlined purposes of research and information of all investigators. As participants were mostly under the age of 18 years, the consent forms were obtained from both themselves and their parents for the students to participate. A total of 39 students agreed to participate in the study. Of these, 9 students were from the low engagement subset, 13 from the moderately engaged subset, and 17 from the highly engaged subset. Due to the presence of more than 10 students from the moderately and highly engaged subsets, they were randomly assigned to two focus groups. Consequently, the study comprised a total of 5 focus groups. Participants were grouped based on their engagement levels because this approach was anticipated to provide a safe environment and to foster richer discussions as participants with similar engagement levels might interact more authentically, facilitating a deeper exploration of their experiences and perspectives (Morgan, 1996). The gender distribution was slightly skewed, with 24 girls and 15 boys. The age range of the participants was between 15 and 18 years, with a mean age of 15.62 (SD = 0.71). More participants were from private schools (*N* = 21) than from public schools (*N* = 18), and the majority of the students were in grade 11 (*N* = 31), with only 8 students in grade 10.

### Data collection

#### Background of researchers

The data collection was undertaken in February 2022. After obtaining informed consent from participants and their guardians, the principal investigator (XZ) conducted semi-structured focus group interviews with a research assistant. XZ was a female PhD candidate with master’s degrees in mental health and applied psychology. The research assistant was a male high school psychology teacher in China with a master’s degree in psychology. Both researchers had been trained in qualitative methods. As the principal investigator and research assistant were implementors of the RCT intervention, both were known by participants prior to focus group interviews. The principal investigator had research interest in mobile health, and adolescent health, she did not hold any assumption regarding the experience of participants as the sample was recruited from different engagement levels. The same with the research assistant. An interview guide was developed by the research team (SE and MB) who were qualitative experts (see Supplementary Datasheet 2) and used during the focus group interviews.

#### Focus group management

Demographic information (e.g., age, gender, and grade) was collected using a questionnaire at the beginning of each focus group interview. The interview guide covered several domains, including participants’ overall experience with using the Coping Camp app, their perceptions of its usefulness for managing stress, and their feedback regarding engagement with the app. Additionally, participants were requested to provide suggestions for improving the usefulness and engagement of the app. All focus group interviews were conducted at psychological counselling centres at two schools during moral classes where students normally engaged with non-academic activities (e.g., watching news on TV). Field notes were made by the research assistant during focus group interviews. Each focus group interview lasted between 45 and 60 min. All interviews were conducted in Mandarin by the principal investigator who speaks both Mandarin and English and were audio recorded with their permission. All interviews were transcribed and translated into English for subsequent analysis. All participants were deidentified by assigning reference pseudonyms.

### Data analysis

The data analysis involved inductive thematic analysis (Braun and Clarke, 2006). It was conducted by two independent analysts (XZ and SE). SE had a PhD degree with a background in telehealth, mental health and education. Initially, all transcripts were anonymized and imported into NVivo 12 software to facilitate coding and theme generation. The analysts began by thoroughly familiarizing themselves with the data through repeated readings, making notes where necessary. Subsequently, initial coding was conducted, followed by the development of a coding framework. The framework was refined through discussions within the research group. A code book was developed and used to ensure consistency between the two analysts. Finally, themes were generated by grouping codes that shared similar content. The resulting themes described the *usefulness of the app* and the level of *engagement with the app*. This was followed by reviewing and refining the main themes and identifying subthemes. In the final step, the subthemes were refined. The analysis began since data collection. Reflective journals were kept throughout the analysis process to reflect how decisions were made about which code to use and the process of generating themes. The reflective journal was reviewed by a senior analyst (BX or MB). Any disagreement was resolved by involving a senior qualitative analyst (BX or MB).

### Ethical review

The study was approved by Research Ethics Committee of Tianjin Normal University in China (2021041901) and Human Ethics Office of The University of Queensland in Australia (HE000791).

## Results

Following our analysis, two main themes emerged: (1) the mechanism and determinants of the usefulness of the app; and (2) facilitators and barriers to the engagement of the app.

### Theme 1: the mechanism and determinants of the usefulness of the app

#### Subtheme 1.1: mechanism of the usefulness of the app.

Most of the participants perceived the Coping Camp app to be useful and relevant for stress management (see Table 1 for themes and subthemes). Overall, participants thought the coping skills training provided by the Coping Camp app was useful in helping them relax during stressful times, improve sleep quality, think more positively, and manage time better. Therefore, the app helped them regulate emotions and reduce stress from homework and study.

**Table 1:** Themes and subthemes.

Themes	Subthemes
The mechanism and determinants of the usefulness of the app.	Mechanism of the usefulness of the app.
	Factors restricting the app’s usefulness in stress reduction.
	Suggestions for improving the usefulness of the app.
Facilitators and barriers to the engagement of the app.	Facilitators of the engagement of the app.
	Barriers to the engagement of the app.
	Suggestions for improving the engagement of the app.

*…I can record my sleep time on my watch. Previously, I only slept for around 5 hours. However, after starting the app's training program for about two weeks, most of my sleep time has stabilized at more than 7 hours. In my opinion, this app is very useful. (p2, moderately engaged group)*

Furthermore, the app was useful for managing stress because of its relevance. The participants found the stressful scenarios presented in the Coping Camp app to be relatable to their daily lives. As a result, they were better able to understand and learn how to manage stressors using the skills taught in the app.

*…There were many stressful scenarios in this app, and I could relate to the characters in the scenarios. I remember that there is a scenario where parents had too high expectations for the children’s exam scores, which caused conflicts, and then we were asked what to do by using the skill learnt in that session. In fact, I had these kinds of problems, but I did not think about how to deal with them before, they just kept happening. After using the Coping Camp, I started to think about using these skills to solve these problems, to face and deal with these problems. (p2, moderately engaged group)*

In addition, students also reported that the discussion board in the app provided a platform for them to share experience, they felt heard, understood and supported by their peers.

*I posted few times on the discussion board when I was under a lot of pressure before monthly exams, some replied to me and gave me support and encouragement, and I felt heard and encouraged. (p6, highly engaged group)*

#### Subtheme 1.2: factors restricting the app’s usefulness in stress reduction

##### Stressor-related factor

While most students found the Coping Camp app to be useful in stress reduction, some students thought the app’s usefulness was restricted when the stressors were unexpected, acute and complex. When students confronted with unexpected and acute stressors, they were usually in bad mood, for example, they might feel angry or annoyed after an unexpected conflict with classmates. Therefore, they reported an inability to use coping skills learnt in the app, instead, they tended to revert to their old ways of coping which were usually maladaptive.

O*ur stressors can be divided into two types, those that resulted from accidental events and those that have existed for a long time. For accidental events, the use of the solutions provided by Coping Camp was very limited. As for the latter, for example, the stress from the long-term existing problems of family or school, the app is useful. (p4, highly engaged group)*

In addition, when the students confronted complex stressors, which were intricate and involved multiple interrelated factors, they found it difficult to apply the skills learnt in the app to deal with these stressors.

*The app was useful most of the time… very helpful in relieving all the stress of the minor things in my life. But for solving some very complicated stressors, it was difficult for me to apply those skills. (p3, moderately engaged group)*

##### Skill-related factor

The study participants reported that they found simple skills, such as muscle-relaxation and mindfulness, easy to master and apply to real-life situations. However, they found that more complex skills, such as cognitive and problem-solving skills, required additional time and guidance to master. Due to school restrictions on mobile phone use, the participants reported that they had limited opportunities to practice these complex skills. Furthermore, they noted that the cognitive load required by these skills often led to frustration and a sense of being overwhelmed, which in turn hindered their ability to practice and master these skills effectively. As a result, the participants perceived the complexity of the skills to be a limitation of the Coping Camp app in helping them manage stress.

*Some skills in the app were relatively simple, we could use them immediately after learning, for example, mindfulness and relaxation skills. However, Coping Camp has many sessions, and all of them were on the phone, and now we still have some difficulties with accessing the phone. There are some skills that we have not mastered very well. Sometimes when we encountered some stressful situations, we cannot remember the skills very well. Generally speaking, the app is still very helpful for alleviating and reducing stress. (p4, moderately engaged group)*

#### Subtheme 1.3: suggestions for improving the usefulness of the app

The students offered several recommendations to enhance the usefulness of the Coping Camp app. Specifically, they proposed the integration of a one-on-one chat feature and a search function to support their management of unexpected and acute stressors. Additionally, the students suggested that the application could provide a platform for planning, monitoring, and tracking their progress in stress management. By implementing these features, the application would potentially provide a more comprehensive and personalized approach to stress management for students.

First, the participants expressed their desire for the Coping Camp app to incorporate a one-on-one chat feature that would enable them to manage unexpected and acute stressful events more effectively. They emphasized the importance of communicating with a human counsellor during times of heightened stress, as opposed to relying solely on automated features of the app. Additionally, participants hoped that the designated chat personnel would be able to offer personalized solutions and practical suggestions for addressing their specific stressors.

*I think the Coping Camp should have real human beings that are able to intervene…that is to say, when we confronted some unexpected stressful events, someone can talk to us, provide suggestions. In this way, the app can be more effective. (p4, highly engaged group)*

Second, the students expressed a desire for the application to incorporate a search function that would allow them to input their specific stressful situations and receive multiple suggested solutions from the application. They proposed that the application would enable them to experiment with each suggested solution and evaluate its efficacy within the platform. This functionality would potentially enhance the application’s adaptability and personalization in addressing individual stressors.

*For example, if I were quarrelling with my classmates now, I can type in: “I quarreled with my friends, what should I do to deal with the embarrassing situation?” and the app can provide me with several solutions. I can choose the solution I like best, and then I implement it and evaluate the effect of this solution. (p2, highly engaged group)*

Finally, the students expressed their desire for the app to aid them in developing stress reduction plans, as well as to monitor and track their progress using objective assessments. Furthermore, they anticipated that the app would offer them a platform for reflecting on their emotional states and behavioural patterns during stress. Lastly, the students expected the application to regularly prompt them for feedback and updates, particularly in cases where they encountered challenges or setbacks through the use of reminders.

*I hope this app can help me record and make plans for stress management. (p6, lowly engaged group)*

### Theme 2: facilitators and barriers to the engagement of the app

#### Subtheme 2.1: facilitators of the engagement of the app

Overall, participants found the Coping Camp app to be engaging because it was novel, the arrangement of content was logical, and it combined psychoeducation with skill practice, involved multimedia and encouraged peer interaction.

First, the students reported that it was their first time to use a mental health app, they found the Coping Camp app to be novel compared with traditional mental health services.

*It was a very novel experience for me, I had not received such training through online platforms before. (p1, highly engaged participants)*

Second, they thought that the arrangement of each session was logical, which made it easy to follow and comprehend:

*For each session, I could still feel that that session had a purpose, a theme and a content. I could also understand its entire process and logic. (p1, lowly engaged group)*

Third, the students also thought that the Coping Camp app was a good combination of psychoeducation and skill practice. Students felt they had the opportunities to learn what was stress, why stress coping skills helped, and to practice those skills. They found this feature engaging.

*I think the app is engaging because the sessions divided the contents into psychoeducation and practice. The psychoeducation helped us understand stress from the beginning of the session, and the theories were had very clear logic. Then it guided us slowly and let us learn to practice and solve the stressors by ourselves. (p3, moderately engaged group)*

Fourth, the students thought that the presentation of content in the app was diverse; it had videos, audios, texts, scenarios and graphs, which made Coping Camp engaging.

*And its functions also included rich presentation approaches, including videos and stressful situations and so on. (p3, moderately engaged group)*

Finally, the students commented that peer influence was an important factor for the engagement of the app. When participants found that their classmates and friends used the app and practiced the skills, they wanted to use the app more often. If their peers knew these skills, they tended to communicate with each other and practice the skills together with their peers.

*If the whole class was interested in this session, I wanted to use the app more often. For example, when we learned mindfulness, we were very excited, when we ate and walked, we said let’s do it in a mindful way. (p3, lowly engaged group)*

#### Subtheme 2.2: barriers to the engagement of the app

First, the students expressed their dislike for text-heavy instructions and their discontent with the number of blank spaces within practice section in few sessions. Such aspects were deemed overly demanding of their cognitive resources, both in terms of comprehension and completion, leading to a tendency to abandon these sessions.

*There were many questions I needed to answer, and I wanted to give up. (p2, moderately engaged group)*

Second, few students expressed a dislike for the pedagogical and monotonous style adopted in educational videos, as they perceived such approaches to be tedious. Rather, they indicated a preference for videos that were engaging, interactive, and humorous in nature, as opposed to the conventional style of didactic lecturing.

*The videos can be a little interactive, for example, you can put multiple choices in the videos…The videos can be more interesting, which will increase our interest. (p5, moderately engaged group)*

Lastly, the students reported encountering connectivity issues and software glitches which hindered their engagement with the application.

*I don’t know whether it was due to the poor internet connection in our school or what, there were times when the videos could not load. (p5, lowly engaged group)*

#### Subtheme 2.3: suggestions for improving the engagement of the app

Participants put forward a series of recommendations aimed at enhancing the engagement of the application. These included the incorporation of gamification features, customization options for the user interface (UI) design, and the implementation of effective dissemination strategies. It was suggested that by integrating these features, the application may become more engaging and appealing to teenage users, ultimately increasing its potential reach and user base.

First, the participants expressed a strong preference for the inclusion of gamification features within the app. Specifically, they suggested that the application should present its content and opportunities for practice in a gamified manner. Additionally, the app should offer incentives as a means of motivating engagement and provide social networking opportunities to facilitate interaction among app users. They thought that the incorporation of such gamification elements has the potential to promote engagement, enjoyment, and motivation, while fostering a sense of community among users.

*For example, we can get some points after we finish each session, and we can exchange these points for some things, for example, things to decorate the app… (p5, moderately engaged group) we can exchange these points for small medals, or small ring made of recycled waste from donation agency…it is very ceremonial. (p6, highly engaged group) …If we can do the tasks in the app as a game, maybe everyone will be more willing to play with it and reduce their stress, learning new skills and playing at the same time…we will like it more. (p3, lowly engaged group)*

Second, the students held divergent views with regards to the design elements of the application, including the app logo and the colour scheme of the user interface (UI). They expressed a desire for the UI to be visually appealing and potentially customizable, with the option to alter the colour of the page and select specific UI features. By allowing for greater customization, the application’s design would better align with the personal preferences of its users, potentially enhancing engagement and overall user experience.

*I mean… the app can be designed to be more interesting…because the icon looks a bit formal now. (p5, highly engaged group) …I think the colour can be more peaceful…when I used it, sometimes, when I looked at the pages, I sometimes felt a little bit sleepy. (p3, moderately engaged group)*

Finally, the students provided suggestions regarding strategies to attract more teenage users to the Coping Camp app. Specifically, they proposed integrating elements that would appeal to students’ interests, such as inviting popular celebrities or bloggers to endorse or recommend the app. By leveraging the social influence of well-known figures, the application could potentially expand its reach and increase its user base among the target demographic.

*For example, find some well-known people, such as to endorse or recommend this app, or…there are many of us who chase stars, if their idol endorse or recommend the app, they would be more willing to use the app. (p4, highly engaged group)*

## Discussions

### Principal findings

This study is the first to investigate user experience and perceptions of a stress management app, specifically for a cohort of Chinese school adolescents. Study results demonstrated that Chinese school adolescents reported a positive experience using the Coping Camp app, finding the app to be relevant and useful for stress management. Also, students described the app as engaging. Specifically, they identified several facilitators and barriers to both usefulness and engagement of the app. They reported that the stress management app was useful in managing chronic and simple stressors, however, its usefulness was limited in managing acute, unexpected and complex stressors. In order to effectively manage these stressors, one-on-one chat and frequently prompt reminders were suggested to be integrated into the app. They also identified cognitive skills and problem-solving skills were complex and thus required more time and guidance in order to effectively using the app to manage stress. They also identified peer support within the app and relevance to their stress experience of the app to be factors that affected the usefulness of the app. With regards to engagement of stress management app, students identified several facilitators of engaging them. These included the form of app intervention itself was novel, the involvement of psychoeducation and skills practice, and positive peer influence within the app. They also identified several barriers to engagement, including text-heavy content, pedagogical and monotonous tones in the videos and few reported confronting connectivity issues and software glitches while using the app.

### Usefulness

Chinese school adolescents who had experience using the Coping Camp app found it useful for stress management. This result is consistent with previous studies that suggest adolescents have a positive attitude toward mental health apps (Tran et al., 2018; Jembai et al., 2022). This study also suggests the potential factors facilitating the usefulness. First, the coping skills training was useful in facilitating somatic, cognitive and behavioural changes, thus leading to emotion regulation and stress reduction. This highlights the importance that stress management app should be underpinned by evidence-based techniques. In addition, the present study also suggests that perceived relevance of the app to the daily lives of adolescents is an important factor influencing its usefulness. For instance, within the Chinese cultural context, emphasize is uniquely placed on academic success and filial piety to parents (Hui et al., 2011). Chinese adolescents reportedly endure significant academic stress and familial expectations and exhibit a propensity to internalize stress and emotions. They often lack adaptive and effective coping mechanisms in response to these tresses (Zhou et al., 2023a). Consequently, it is critical that stress scenarios incorporated in the app are reflective of the school environment and familial expectations, and employ evidence-based strategies optimized to alleviate such distinct form of stress. Also, similar to adolescents in other cultures (Dijk et al., 2007; Ellis et al., 2009; Ali et al., 2015), peer support is valued by Chinese adolescents to be a facilitator for reducing stress in self-help mobile app, this is in line with previous finding suggesting the integration of peer support in mental health apps (McColl et al., 2014).

Also, this study indicates the stress management app is more useful in managing simple and chronic stressors, however, its usefulness is limited in managing unexpected, acute and complex stressors. It is worth noting that the students did not distinguish between different sources of stress (e.g., academic, interpersonal, or family-related stressors) when evaluating the usefulness. This has important implications for self-help management apps, highlighting the necessity to integrate real-time support from mental health professionals (e.g., one-on-one chat) in such apps to comprehensively address perceived stress among adolescents. Additionally, students thought that complex skills were more difficult to master therefore limited the usefulness of stress management apps. This highlights the importance of incorporating more guidance and more effective methods to deliver such skills in mobile apps. Moreover, earlier research has indicated the effectiveness of utilizing monitoring apps for addressing mental health issues (Wang et al., 2018), the present study contributes to this body of knowledge by demonstrating that adolescents exhibit a preference for monitoring features combined with planning capabilities within such apps to facilitate the acquisition of coping skills.

### Engagement

Previous studies have reported low levels of engagement in mental health apps (Connolly et al., 2021; Melcher et al., 2022). This study contributes to the existing literature by identifying factors that impact adolescent engagement in such apps. Firstly, in line with previous studies suggesting young people prefer simplicity and customizability in mental health apps (Liverpool et al., 2020; Melcher et al., 2022), Chinese adolescents appreciate a well-organized, straightforward arrangement of app content, and customizable UI design. Secondly, for stress management apps, the study found that combining psychoeducation with coping skills practice is essential in helping users understand and effectively practice stress management techniques. Thirdly, in line with a previous study suggesting adolescents prefer videos, limited text, ability to connect with others within mental health apps (Liverpool et al., 2020), present study also suggests that presenting content in various formats, such as videos, audio, texts, and graphs, is deemed to be an effective way of making the app engaging and interactive. Fourthly, peer influence has been suggested by literature to play an important role in adolescents’ behaviours (Gifford-Smith et al., 2005; Prinstein and Dodge, 2008), present study suggested that peer influence is also a significant facilitator in utilization of mental health apps, highlighting the importance of considering this factor in the development of mental health apps. Finally, previous systematic reviews suggest that gamification features were not effective in managing mental health problems (Six et al., 2021) or enhancing adherence (Cheng et al., 2019; Six et al., 2021) among general population. However, present study suggests that the adolescent population prefer gamification features or game-like designing (i.e., serious games) in stress management apps, indicating such features may be effective in enhancing engagement and health outcomes, thus should be considered in the development of stress management apps.

Our study identified several potential barriers to engagement with the Coping Camp app. First, even though previous reviews suggest that text-based interventions are acceptable and feasible in delivering synchronous counselling services (Hoermann et al., 2017; Dwyer et al., 2021; Zhou et al., 2022a), present study suggest that text-heavy content is likely to disengage adolescents in self-help mental health apps. Second, this study also suggests that adolescents prefer interactive, humorous and friend-like tones to pedagogical and monotonous tones in psychoeducation videos. Last, connectivity issues and software glitches were identified to be disengaging, indicating that these barriers should be addressed while designing stress management apps for school adolescents.

The outcomes of this study highlight several practical implications. Firstly, given the scarcity of mental health professionals in school settings in China, it is pertinent to consider the utilization of mental health apps, which are reportedly well-accepted by adolescents, as complementary resources for students. Secondly, the development of stress management apps for adolescents necessitates the integration of human therapists to assist those facing acute and complex stressors, ensuring comprehensive support. Moreover, to encourage sustained use, the content of these apps should be concise, engaging, and user-friendly for adolescents.

### Strengths and limitations

This study has several strengths. Firstly, all participants had practical experience using the Coping Camp app, thereby lending credibility and practical insight into app development based on real-world experience. Secondly, the study’s participants were representative of the intervention arm within the randomized controlled trial, covering diverse engagement levels, both genders, and those attending both public and private schools. Nevertheless, a few limitations should be noted. Firstly, participant recruitment was not based on stress levels, hence the study cannot accurately represent individuals with different stress levels, who may possess differing perspectives and preferences for stress management apps. Secondly, the results of this study may not be generalizable to stress management apps that utilize different psychological techniques (e.g., acceptance commitment therapy, positive psychology) or other modes (e.g., chatbots, serious games).

## Conclusion

This study demonstrates that Chinese school adolescents considered using stress management apps such as the Coping Camp as positive and satisfactory experience. The app was useful for stress reduction through facilitating positive somatic, cognitive and behavioural changes. Relevance to their real-life stressful situations, provision of peer support, integration of planning and monitoring features, and simple and straightforward coping skills were considered to be useful features for stress management apps. In addition, present study suggests its usefulness may be limited when managing unexpected or acute stressors. The integration of real-time support via one-on-one chat with human professionals might facilitate its usefulness in managing such stressors. This study demonstrates that multimedia, logical arrangement of contents, combining psychoeducation and skills training, positive peer influence, gamification features, customizable and appealing UI design appeared to be engaging adolescents, whilst text-heavy content, pedagogical and monotonous tones, and connectivity issues and software glitches appeared to disengaging adolescents in stress management apps. Future studies should consider these factors while developing stress management apps for adolescents.

## Data availability statement

The data analyzed in this study is not publicly available, due to ethical restrictions to protect participant privacy. Requests to access these datasets should be directed to XZ, xiaoyun.zhou1@uqconnect.edu.au; xiaoyun.zhou@uq.edu.au.

## Ethics statement

The studies involving humans were approved by Research Ethics Committee of Tianjin Normal University in China (2021041901) and Human Ethics Office of the University of Queensland in Australia (HE000791). The studies were conducted in accordance with the local legislation and institutional requirements. Written informed consent for participation in this study was provided by the participants’ legal guardians/next of kin.

## Author contributions

XZ contributed to the conceptualization, methodology, data collection, formal analysis, writing—original draft preparation, played a leading role in conceiving the research question, developing the research methodology, and drafting the original manuscript. MB was primarily responsible for performing the formal analysis, contributed to the conceptualization, methodology, formal analysis, writing – review and editing, supervision, reviewing and editing the manuscript, ensuring the analysis was accurately represented, provided supervision for XZ. XB was primarily responsible for performing the formal analysis, contributed to the conceptualization, methodology, formal analysis, writing – review and editing, supervision, reviewing and editing the manuscript, and provided supervision for XZ. AS contributed to the conceptualization, methodology, participant recruitment, writing – review and editing, supervision, participant recruitment, together with reviewing and editing the manuscript, provided supervision for XZ. SE contributed to the conceptualization, methodology, data analysis, writing – review and editing, supervision, formal analysis, reviewing, and editing the manuscript and provided supervision for the whole project. All authors contributed to the article and approved the submitted version.
