# Supplementary material for: Exploring factors affecting Chinese adolescents’ perceived usefulness and engagement with a stress management app: a qualitative study
Source: Front Psychol. 2023 Nov 20;14:1249093. doi: 10.3389/fpsyg.2023.1249093 (PMC10694271; doi:10.3389/fpsyg.2023.1249093)
Supplement: Supplementary file 2 [file Data_Sheet_2.docx]

**Article title**: Exploring factors affecting Chinese adolescents’ perceived usefulness and engagement with a stress management app: a qualitative study

**Journal name**: Current Psychology

**Author names and affiliations**:

Xiaoyun Zhou^1,2^; Matthew Bambling ^3,4^; Xuejun Bai^5^, Anthony C. Smith^1,2,6^ and Sisira Edirippulige^1,2^

**Corresponding author’s email**: xiaoyun.zhou1@uqconnect.edu.au

**Interview guide**

Q1: What was your experience with using the Coping Camp app?

Q2: Do you find it helpful in managing stress? In what aspects do you think it is helpful in managing stress?

Q3: Do you find it unhelpful? In what aspects do you think it is unhelpful?

Q4: What do you like about the Coping Camp app?

Q5: What do you dislike about the Coping Camp app?

Q6: In your opinion, what changes should be made to the Coping Camp app so that the app could better help you reduce stress?

Q7: In your opinion, what changes should be made to the Coping Camp app so that the app could better engage you?
